# Placing precarity: access and belonging in the shifting landscape of UK mental health care

**DOI:** 10.1007/s11013-020-09683-5

**Published:** 2020-11-06

**Authors:** Natassia F. Brenman

**Affiliations:** Institute of Public Health, University of Cambridge, Cambridge Biomedical Campus, Cambridge, CB2 0SR UK

**Keywords:** Precarity, Mental health, Access to care, Migration, Sociomateriality

## Abstract

This paper engages with the notion of ‘embodied belonging’ through an ethnography of the social and material aspects of accessing mental health care in the UK. I focus on moments of access and transition in a voluntary sector organisation in London: an intercultural psychotherapy centre, serving a range of im/migrant communities. Whilst both ‘belonging’ and ‘place’ are often invoked to imply stability, I explore how material contexts of access and inclusion can paradoxically be implicated in the ongoing production of precarity—of unstable, uncertain, and vulnerable ways of being. A sociomaterial analysis of ethnographic material and visual data from two creative mapping interviews attends to material and spatial aspects of the centre and its transitory place in the urban environment. It demonstrates how these aspects of place became entangled in client experiences of access: uncertainties of waiting, ambivalence towards belonging to a particular client group, and questions around deservingness of care. This engendered an embodied and situated experience of ‘precarious belonging’. I therefore argue that precarity should be ‘placed’, both within the concept of embodied belonging, and ethnographically, within the material constraints, impermanence, and spatial politics of projects to include the excluded in UK mental health care.

## Migration: A Clinic in Transition

### 13th September 2016

As it stands now, the psychotherapy centre I call Culture in Mind operates from a tiny, solid building in inner city London, squashed between an express supermarket and a Lebanese café, whose tables and chairs spill onto the pavement outside. It is so close to the bus, train, and underground station that you can hear the tannoy announcements from behind the frosted glass that separates the inside of the centre from the street. But this is all about to change; the staff have known for some time now that they would not be able to stay in the current building for long. The rent is being hiked to almost double what it is now, and everyone agrees that—even if they had raised the money to keep up with the higher rent—it wouldn’t have been worth it to stay. The therapists have been saying for years that the windowless rooms are unacceptable for the kind of work that they do in the centre: it is not unusual for their clients to have been through incarceration, interrogation, or even torture. This was shaping much of the decision-making about the building work that was needed to convert a new place in a neighbouring area from an office space to a therapy centre.

This explains why, on an unusually hot September day, I am leaving the current site of the therapy centre with some of the staff to take a bus to the new building, about a mile and a half away. The two therapists I am with are discussing the building work with the clinic’s director. They are worried that costs are being cut in the building of doors to each therapy room, which need to be private but also designed so that vulnerable people are never completely out of sight. “We can’t have *spyholes*!” one of the therapists, K, reiterates, looking fierce. “It will create an atmosphere of suspicion”. The conversation continues, and more dilemmas are raised about the tight budget and the resources needed to create a proper therapeutic space. Before long, the bus stops in leafier, more residential environs than we have just come from and we find ourselves standing outside a block of seventies-style ex-office buildings. We enter a lobby area and stairwell, which we are enthusiastically told by the site manager could be used as a waiting room. Opening a second set of doors into what would become the clinic space, we are hit by a wall of cool air-conditioning and a strong smell of gloss paint, before we walk in and see the newly partitioned therapy rooms.

The clinic-in-transition I describe in this scene is a voluntary sector mental health service in London, which serves a range of minority ethnic and im/migrant^1^ communities in its provision of intercultural, multi-lingual psychotherapy. Some clients are fully established in the UK and the diverse neighbourhoods the centre serves, but many are new migrants or refugees, reflecting the “super-diversity” of London’s different communities; the accumulation of many waves of migration and demographic change (Vertovec 2007; Hall 2013). Several transition stories operating at different scales collapsed in on this twenty-minute period of movement: potential client journeys to the new therapy centre, the logistics and politics of the organisational move, and the spectre of transnational migration—of interrogation rooms and unfamiliar institutional settings.

This paper attends to such coexisting practices of inclusion and exclusion but focusses on inclusion beyond legal status, looking to the “micro dimensions of therapeutic geographies” (Parkinson and Behrouzan 2015:326). This is particularly significant in an era of austerity and increasing decentralisation of mental health care from the state to voluntary services in the UK. These services are often in a better position to reach out to those who cannot access or do not ‘belong’ in other parts of the care system (due to migration status or otherwise), but there are concerns about overreliance on a sector that often lacks the material resources to provide spaces of stability (Nellums et al. 2018). Echoing what Fassin and Rechtman elegantly summarise in their genealogical account of migration and mental health care in France, “the price of liberty for these initiatives is their marginality” (2005:354). At stake here are the material and spatial limits of access and inclusion; values that are central to the logics of the voluntary care sector. Foregrounding the sociomaterial aspects of belonging, or ‘being in place’ in mental health care (Pols 2016; Ootes et al. 2013a, b), I ask: how do places of care *participate in*, *act on* and *enact* belonging or non-belonging within voluntary sector mental health care?

I engage with the notion of ‘embodied belonging’ (Mattes and Lang, this issue) through asking these questions about place, materiality, and belonging in the context of my ‘ethnography of access’ in UK mental health care. In this project, my material and conceptual focus is around the ‘doors’ or threshold spaces of these services, rather than the therapy rooms and work that took place within them. When considered as an abstract category in relation to access to care, ‘belonging’ has to do with eligibility, entitlement, and being ‘in the right place’ according to certain inclusion or exclusion criteria. But thought about in terms of the *embodied*—the sensorial, material, and the spatial—belonging becomes something rather different. As Mattes and Lang (this issue) have proposed, belonging becomes both a process and outcome of relations with place, amongst other political and socio-emotional entities. I am particularly interested in what a methodological engagement with embodiment might do to help think through being ‘in-place’; not just in terms of nationality, immigration status, or fitting in within the health care system but rather from the ground up: as a product of relationships with space, materials, and psychotherapeutic care.

In this ‘bottom up’ approach to belonging, these ethnographic data reveal something else about the notion of belonging in current configurations of care provision: that it is inherently *precarious* (Tsing 2015; Allison 2013; Matza 2018). As I move through the ethnographic material and discussion section of the paper, precarity emerges as a central aspect of embodied belonging in this context. Thus, my ‘bottom up’ approach encompasses the notion of precarity, echoing recent work that has explored precarity, “ethnographically as a situated, processual condition that emerges in urban assemblages” (Bieler and Klausner 2019:209). Building also on the ethnographic scholarship of anthropologists who have explored ambiguities and paradoxes in the notion of belonging (Stevenson 2014; Lien 2015), I seek to understand the relationship between states of belonging and precarity, as they emerge in the material context of my field site, one of three psychotherapy centres I carried out ethnographic fieldwork in between 2016 and 2018.

This paper therefore aims, firstly, to describe a particular case in which access to mental health care is a key concern; exploring how the politics, values, and materialities of inclusion play out at the intersection of mental health care and migration. Secondly, it seeks to provide fresh insights on questions of access and inclusion by focussing on the material and spatial aspects of belonging for two women I came to know at the centre. For this, I employ a creative interview method I developed to generate data on ‘moments of access’ at this site (White, Hillman, and Latimer 2012). Finally, it provides an analysis of the ambivalent, at times paradoxical, nature of embodied belonging, engendered by the social and material context of voluntary, extra-state care. Namely, that a sense of precarity is always to some extent embedded within belonging, but that this sense of precarity becomes more overt in particular places in the landscape of UK mental health care. The contribution of this paper is therefore to ‘place’ precarity, both within physical spaces of care, and at the heart of the concept of embodied belonging.

## The Politics of Access and Inclusion

Before turning to the creative method that I employed to explore the more grounded, embodied notion of belonging I have touched on above, I start (as I did in my ethnographic fieldwork) with more structural questions of belonging. These relate to the politics of *access* to care and non-governmental projects aimed at *inclusion*. First, I provide some background to the politics of access in my field site before discussing the (now well-rehearsed) ways in which access and inclusion have been shown to be tightly and relationally bound to exclusion in health care (Parr 2000; Willen 2012b; Lähdesmäki et al. 2016). I then go on to explore a specifically ‘sociomaterial’ lens through which similar paradoxes might be explored and new insights generated about belonging ‘in-place’.

The centre from which I draw my data for this paper is one of a small cluster of voluntary sector organisations I worked with, which provide psychotherapeutic care to people who, for various reasons, have limited access to mainstream or private care. These organisations have been doing so for many years but the imperative for providers to compete for funding to carry out their work (in this case, tackling problems of access to care and unmet need) has intensified in the current system of commissioning services in the UK. Following the implementation of a long period of austerity, the introduction of the “any qualified provider” policy of the Health and Social Care Act (2012) made explicit its reliance on voluntary services to provide care to “underserved and specialised groups” who find it harder to access services via mainstream clinical routes. Voluntary organisations now bid for short-term contracts with an increasingly fragmented National Health Service, which will fund services according to their efficiency and ability to demonstrate the need for these services in communities. This centre had managed to secure their contract and other forms of funding in part because of their focus on providing intercultural, mother-tongue therapy to migrant and refugee groups, who were at that time highly visible as vulnerable groups with low access to care (Fassil and Burnett 2015).

It is here, at the policy level, that the links between (good) mental health, access to care, and a sense of belonging are laid out (Namer and Razum 2018). This reflects the literature on migration and mental health where belonging is often referred to as something to be achieved in a post-migration context (Kirmayer et al. 2011; Castañeda 2010) and so is often intuitively associated with ideas of being settled, in-place, or “at home” (Yuval-davis, Wemyss, and Cassidy 2017). However, in practice, the achievement of belonging through accessing care and being included in service provision is rarely straightforward; moreover, it is tightly and relationally bound to exclusion at multiple different scales.

Access to the Culture in Mind centre was not a case of belonging to particular ethno-cultural community, or even falling into the category of ‘migrant’; rather, inclusion criteria were defined in relation to an absent majority or mainstream that was understood to be imbued with Whiteness (Brenman 2019a). For this reason, service users and service providers could not be clearly delineated according to defining features such as cultural background, migration history, or immigration status. The therapists, staff, and volunteers all identified as coming from different ethnic or cultural minorities, and many were first-generation migrants or refugees. (The reason that K had been so appalled by the idea of installing “spyholes” to the therapy room doors was that she was herself a political refugee from an oppressive state system). The therapeutic intercultural approach developed in the centre was therefore infused with a postcolonial, anti-racist politics (cf Giordano 2014, on ethnopsychiatry and migrant mental health in Italy). Crucial to the initial establishment of the centre was a political acknowledgement that ‘belonging’ has not historically been something associated with psychotherapy settings (or mental health care more broadly) especially for those who do not fit the traditional moneyed, white, middle class, and (in the UK) English-speaking archetype (Lipsedge and Littlewood 2005; Kareem and Littlewood 2000). Reflecting the trade-off between “liberty” and “marginality”, which was observed by Fassin and Rechtman (2005) in the French psychiatric system, the work of inclusion here was always shaped by wider practices of exclusion.

Ethnographic scholarship has in various ways thought through problems of inclusion of the ‘other’ in this field (Ticktin 2006; Fassin 2012; Willen 2012a; Cabot 2013, 2017). Cabot has, for example, raised concerns about instances of collective openness to migrant ‘others’ in the form of xenophilia (love for or attraction to the “stranger”) because of the inherent *instability* of such a mood—a mood which may reinforce distinctions between self and other, only to then shift in direction (Cabot 2017). More specifically to the issue of access to health care, Willen (2011) has interrogated the seemingly unproblematic notion of the “right to health”, building on the theoretical arguments made by Greco (2004) about the assumptions this notion carries about the singularity and stability of ‘health’ and ‘rights’. Crucially, Willen’s analyses tell us that in *practice*, rights to health and inclusion within spaces of care can become blurred, for example, through moral judgements of deservingness (2012a, b). Other ethnographers too have critically considered practices of giving and receiving care in charity and humanitarian settings, which might initially appear to be unproblematically including the excluded (Gottlieb, Filc, and Davidovitch 2012; Huschke 2014).

The work I have described in this part of the paper usefully interrogates the moral and ethical dynamics of inclusion, showing us how it can be conditional and unequally distributed, even amongst the people these projects are aimed at (Sargent 2012). But these studies tend to frame questions of belonging in moral or ethico-legal terms, whilst the story I have begun to tell about the centre-in-transition, and the clients who would go on to encounter it, takes us a step sideways from this political and legal realm. Particularly when considered through the lens of *embodied* belonging, this story calls for a rather different set of analytical questions about its material and spatial conditions. In the following section, which incorporates my methodology, I introduce a ‘sociomaterial’ approach to thinking about care settings that explicitly foregrounds these conditions.

## Sociomaterialities of (Non-)belonging

Notwithstanding the specific critiques I have discussed above, ‘belonging’ is often invoked in the literature on migration and mental health to imply a sense of stability. This stable state of belonging is also an abstract category, an imagined state of ‘being in-place’. However, this abstraction can be misleading. Lien and Melhuus (2007) introduce their volume of “ethnographies of knowing and belonging” with a cautionary note on imagined belonging: that it has the tendency to unduly ‘fix’ the relations between people and places. In her own work on relations between Tasmanian trees and people, Lien (2007, 2015) seeks to bypass these imaginings about how migration and indigeneity ought to be, by engaging directly with the landscape. This allows for an exploration of questions about belonging in a postcolonial context (in this case, Australia) through material engagements with the country’s ‘nature’. Following the work of Lien and others, I draw on a set of conceptual ideas about sociomateriality in Science and Technology Studies (STS) and non-representational theory, based on the assumption that material and socio-cultural entities come into being in relation to one another (Law 2004; Moser 2017; Thrift 2004).

Considered as a sociomaterial entity, ‘place’ has been usefully employed to understand belonging in health care. Whilst there have been important contributions in this field that highlight the specific *intangibility* of many barriers to health care for im/migrant groups (Larchanché 2012), ‘belonging’ can also be understood as a product of relations between people, spaces, and materials, rather than an abstract category. This emerges vividly in the work of Ootes, Pols, and colleagues, who have considered questions of integration as part of a broader project about care and citizenship for people with long-term mental health problems (Ootes et al. 2013a, b; Pols 2016). Through ethnographic engagement with Dutch de-institutionalised care systems, they have developed a “way of studying citizenship that looks at the relationships between people and the way these relationships are materially mediated and form social spaces” (Pols 2016:178). This engages empirically with the idea that “places can enact relationships” in health contexts, referring to Latour’s assertion that places and objects have agency of their own (Ootes et al. 2013b:16; Latour 1987). Place can therefore be treated as a sociomaterial entity that has powerful potential to affect the social world. Responding along this line of enquiry raises important questions about the ways in which places *participate in*, *act on*, and *enact* belonging or non-belonging within mental health care.

In order to answer such analytical questions, it was necessary to develop a way of accessing spatialised experiences and (re)engaging participating service users (or ‘clients’, as they were referred to in this setting) with their interactions and spatial practices within my field sites. As part of a broader ethnographic approach that focused on practices and processes of accessing mental health care in the voluntary settings I have described above, I employed a creative interview method of visual mapping (Gauntlett and Holzwarth 2006; Literat 2013; Bagnoli 2009; Dennis 2016), which is especially useful for eliciting experiences of a spatial and material nature (McGrath and Reavey 2013). Subverting associations with mapping as a colonial practice of representing territory, I had in mind Ingold’s conceptualisation of the sketch map when developing this method; where there may be no borders separating inside from outside the map, and lines drawn are formed to re-enact a walk along and through terrain (2006:27). The ‘walk along’ in this case is the practice of accessing care: an encounter between person and place. So, rather than asking clients about what they *felt* or how they *experienced* their first encounter with the service, I asked them to draw a map of the centre focusing on first impressions and memories of *what happened* when they accessed the service as a new client.^2^ My approach to ‘the embodied’ therefore attends to more than experience within individual bodies, and tries to think about how *various bodies and materials* make up places and the experiences that emerge in the data (Dennis 2016). In the next section of the paper, I trace the visual accounts of what happened when two of my interlocutors, whom I call Mariam and Dayo, accessed the service. Both produced visual maps that focused intensely on the material and spatial elements of waiting room of the centre, though their experiences of accessing this space contrasted in significant ways.

## Mapping Precarious Belonging

Mariam, a client I had met some weeks before, had agreed to meet me for an interview to tell me more about when she first accessed the service. Because she lived a long way from the centre, I’d offered to travel to her for an interview, but she’d quickly refused, saying she did not want me to visit her home. Although she had moved out of the temporary accommodation she’d been in when I first met her, the house that the council offered her had a severe damp problem and leaked from the roof. Despite all of this, Mariam—originally from Eritrea—was glad she came to the UK, where she claimed asylum status two years ago. She had been trafficked across the Middle East by her ex-husband’s family after leaving Ethiopia where she grew up, believing she was going abroad for the opportunity to study. This time had been intensely violent, leaving her with complicated health problems. She was often unable to sleep and at times she was overwhelmed with sadness, though she didn’t want to take medication for either of these things, which was one of the reasons she was referred to Culture in Mind for talking therapy. In the end, we met back in the centre, in one of the therapy rooms, under a tall plastic lamp, separated by a low table, from which I’d cleared the customary box of tissues and ticking Ikea clock.

## Mapping: Mariam (“Imprisoned” on the Threshold)

### 18th January 2017. The Meeting Room at the Centre

**Fig. 1:**
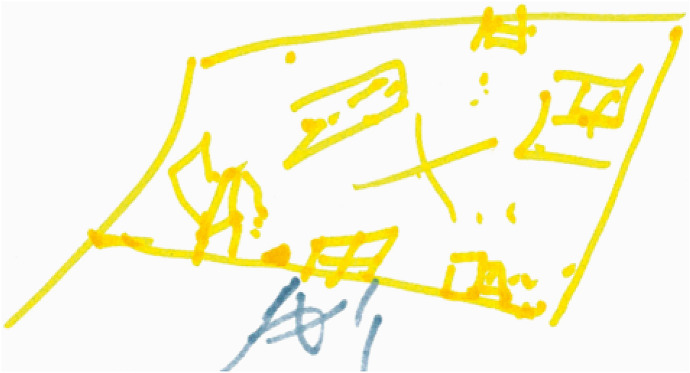
A fragment of Mariam’s map (the waiting room)

Mariam has finished drawing her map (Fig. 1) and we are talking about some of the things it has brought up. She is pointing to the part with her birds-eye view of the waiting room: four walls, a table, a chair standing on its own, and a big cross in the middle, which shows that here, “you don’t have *nothing*”. She has drawn the place swiftly, impatiently, with ongoing commentary about her discomfort at its sparseness. The last part she drew was the inside door, the “secretive” one, leading into the reception and clinical part of the centre, with an oversized blob next to it: the buzzer, which acted like a security guard, stopping one from leaving the waiting room until called. It was this that made the waiting room feel like a prison for Mariam. She tells me that she can never relax whilst she is waiting to be called in. She had drawn this area in yellow, because “yellow means emergency”.

Having set out a particular place within the centre, Mariam now begins to bring herself into the scene in real time. “Here, it is…” She looks sideways suspiciously as though at other clients in the waiting room. Again, she points to the map—to the wall where the rest of the seating is—setting the scene of two other people sitting there looking at her. Then she takes on the role of one of these women, looking back suspiciously whispering incoherently: “sschp sschp sschp!” and then, “she’s… I think she’s—” Mariam does the face of the suspicious woman, suddenly shocked, scared of something, and then bursts into laugher at the thought of this before going back into role. Now, the suspicious woman is looking across the waiting room at Mariam again murmuring: “is she working here? No? Then why is she coming here? What’s her problem?” There is a comical back-and-forth of Mariam playing the two women trying to suss each other out by looking at the other, then quickly turning their heads away. Then she does the suspicious woman saying louder, “Why are you looking at me? Are you going to beat me?!” and then, with a sharp intake of breath, Mariam is herself again, telling me with a laugh that this goes on until suddenly the door opens and she is called in. Only then she can finally leave this strange, tense encounter.

This one-woman role-play that sprung up out of the mapping showed vividly the interactions between Mariam, the other clients, and material aspects of the waiting room itself. She had made the comparison between the waiting room and a prison to me before, but her mapping out of the experience of waiting here enlivened the otherwise fairly generic metaphor, showing how specific ‘things’ (and a lack of things) had participated in this experience and evoked such a powerful and enduring association. She didn’t blame the other women who had seen her and been suspicious of her invisible but potentially frightening, or even dangerous, psychological issues. In fact, she later tells me that they are “just like [her], and [she is] just like them”. But it is not just that the women were performing these subjectivities *in* a shared social space, the place itself—the waiting room—was entangled in the experience. *It was the door with the buzzer* that trapped them in there together (there was no ‘real’ security guard in its place) and the big open space in front of them failed to provide distraction or a buffer from the suspicious looks shooting back and forth between the chairs backed up against the walls. The lack of social cues and activities to occupy oneself with (making it unclear even who was a client and who worked there) is characteristic of liminal places, designed only to wait in. It was also down to the fact that the centre had only recently relocated (a transition in itself) and there simply was not very much to soften or fill this empty area between the clinical space and the outside.

Mariam’s sense of uncertainty and vulnerability was bound up with *this particular* liminal place, as she described an entirely different feeling when she was buzzed into the main space and joined her therapist, with whom she felt extremely safe and at ease. Here, belonging and non-belonging were particularly, and uncomfortably, close. Despite having demonstrated eligibility and already ‘gained access’ to care, this remained mediated by technologies of containment and security. The therapeutic space was momentarily obscured and made conditional even though she knew access had been granted and was imminent.

Multiple and sometimes contradictory forms and metaphors emerged through the mapping of places, objects, and spatial practices within one centre. Where Mariam conjured up the image of a prison, Dayo, another young woman who accessed the service, talked of the centre as being a kind of sanctuary. Dayo’s problem, in *this setting,* was that she didn’t feel like it was *her* sanctuary. When I first met her, whilst she was still coming to the centre for sessions, she had been angry and frustrated with her whole experience of accessing psychotherapy. Her response had been due to lots of reasons but particularly because of the excruciatingly long wait and multiple assessments she had been through between presenting her problem to doctor and getting through the doors of this particular centre. After the months she spent waiting for support—a period she called “being in a black hole”—she had found it hard to feel like anyone was on her side, even once she had started her sessions.

When we met again for another interview some weeks later, she was still resentful of what she had been through, but the anger had dissipated. She took me to a café in a corporate hotel near her house, with a plug socket next to every table for laptops, and a station for adding extra cinnamon or soya milk to your coffee. Reflecting on how the centre had appeared to her, she talked of it being a kind of sanctuary for people from different backgrounds and cultures. But Dayo, originally from Nigeria but completely at home in the UK, had always had a strong sense of being in the *wrong* place in this intercultural therapy centre. Her migration story was one of regular international travel with her father’s company when she was growing up between Africa, Europe, and America, before settling in the UK for a career in investment banking, which she’d recently been signed off from with chronic illness and severe depression. Was this the life of a ‘vulnerable migrant’? How vulnerable was she, really? These were questions that Dayo herself was constantly preoccupied with during her time at the centre.

## Mapping: Dayo (in the Wrong Place?)

### 24th January, 2017. A Hotel Cafe Close to Dayo’s Flat

**Fig. 2:**
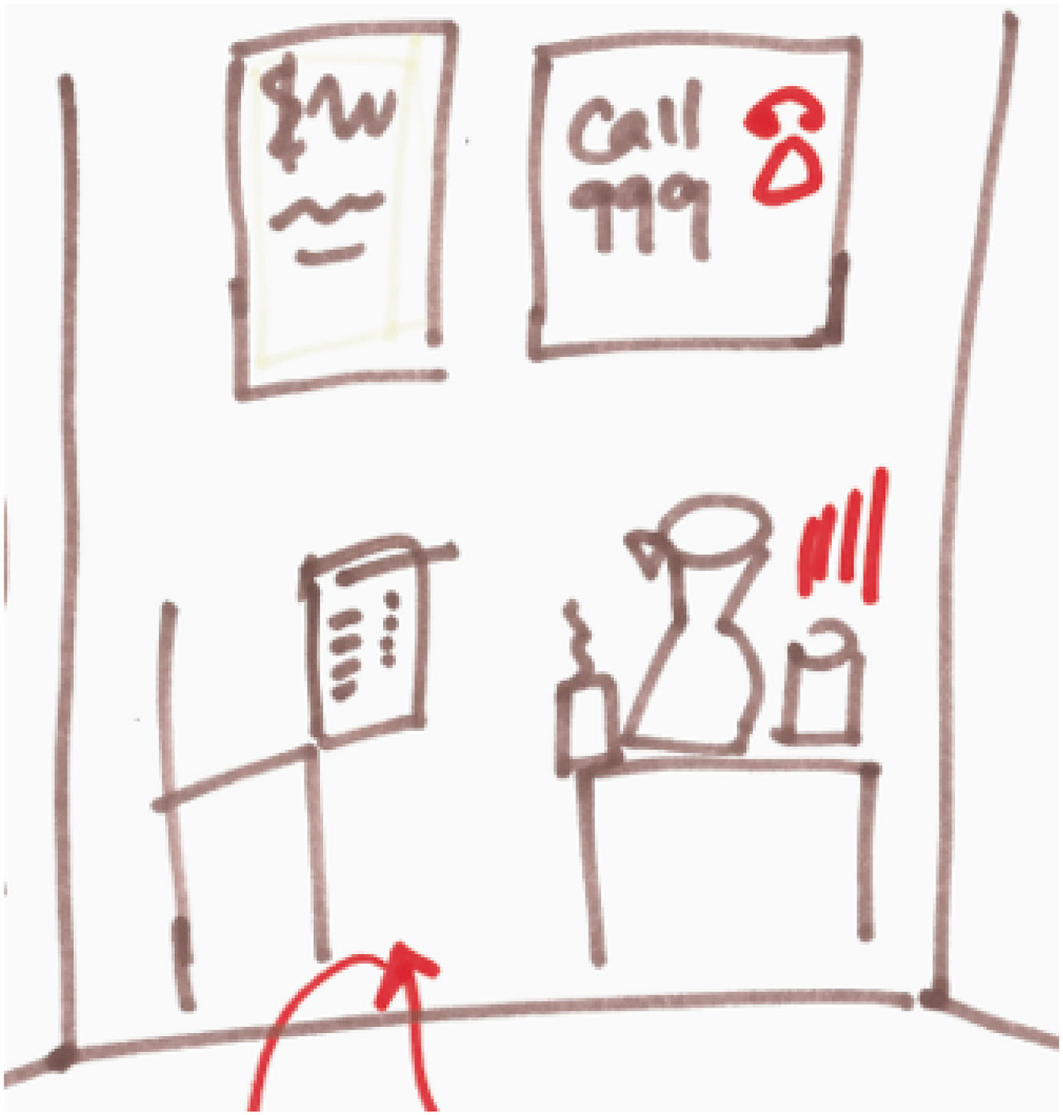
A fragment of Dayo’s map (the waiting room)

Dayo draws out three sections in broad felt-tip strokes, before choosing the middle section to work in Fig. 2. This is her waiting room. The space rapidly fills with illustrations of objects and spatial features of the room, which she narrates as she goes. On top of the outline of the table appears a box of tissues, a jug, and a cup. She says hollowly that this had been hilarious to her: “the classic waiting room, with the water and the tissues. ‘Get crying’”, the objects seemed to have been saying to her. Apparently, these items had appeared cartoonish to her even in ‘real life’, before she had transformed them into two-dimensional motifs on the page. She had produced an image of them as stereotypes as soon as she walked into the room, and, quick off the mark, mocked them rather than falling into role as another stereotype: the vulnerable patient. But the pressure was on, because they weren’t just there to be looked at, or even used if needed. They were *interacting* with her, expecting (demanding?) something of her: to be vulnerable, to “get crying”. She moves on quickly to drawing the posters that she had seen on the walls, placing them full frontally towards us in the middle of the page. One has squiggles on that could be writing but we can’t read it because, she tells me, it is supposed to represent a language she doesn’t understand. The multi-lingual signage, which the centre is careful to display in order to communicate with as many of their client group as possible, had made her think the centre must be for women who were in the UK but dealing with issues from “their own culture”, a different culture. For the first time of many, Dayo quotes the line that had been going round and round her head: “Why am I here?” The other poster has a big ‘CALL 999’ on it and a picture of a telephone, which she later colours in red to explain the sense of alarm it had given off. She knew that the posters were not meant for her. She felt sure that her therapist would think: “what have you got to worry about? Get out of here!”.

Paradoxically, then, Dayo did feel she occupied a precarious position here; her not being vulnerable *enough* made her undeserving of care here—or so she felt. Whilst she believed the place was *supposed* to be a “sanctuary”, she felt like an outsider and an imposter. Not only did she feel like she didn’t really belong, or want to belong there, she almost felt she would be expected to leave. Her vulnerabilities were hidden under a layer of perceived un-neediness and un-deservedness compared to the imagined group the service was *really* for. No one had told her this, but it was what she had taken from her surroundings. The *things* in the room were contributing to her sense of being *in the wrong place*. This is not to say that the tissues and water were to blame for Dayo’s insecurities about belonging and deserving care, but in the moments of physically accessing of the service, these objects were enlivened by the dynamics at play between a particular person entering into a particular space. Although very different to Mariam’s story, I saw parallels in the way that this waiting room space became an active medium in producing their experiences of accessing care.

## Placing Precarity

These accounts are best understood in the context of the clinic itself, as well as the personal lives of Mariam and Dayo. These ‘moments of access’ (White, Hillman, and Latimer 2012) were embedded in the ongoing practicalities and politics of the clinic-in-transition that I introduced at the beginning of this paper. In addition to the changing position of the voluntary sector in relation to the mainstream mental health system (playing an increasingly major role in an increasingly fragmented system), this clinic was of course *physically* and *geographically* moving to a new site. Mariam and Dayo had encountered the clinic during this period of transition, and the therapists were far from unaware about what effects of the move may have on current clients. In this section, I ‘place’ precarity ethnographically, within the shifting landscape of UK mental health care.

Moving day had taken place on a Saturday after weeks of anxious packing, labelling, and (attempts at) preparing clients for the upheaval. Slowly, and true to their commitment to cultivating a sense of belonging, the staff worked to make the place as homely as they could with effectively no budget; pot plants were brought in and the kitchen supplies built up as every week a different pair would cook for everyone (bowls, seasonings, and a healthy supply of leftovers quickly accumulated over time). In those early weeks of the new place, when everything was so new and our surroundings so present and visible, there was much talk about which pictures should hang where, which parts of the clinic should be open or closed, and what it meant for the clinical parts to be “therapeutic”. In this way, questions of inclusion and difference came to be discussed through these mundane material practices. Crucially, values of accessibility, inclusion, and belonging were not givens; they had to be made and re-made and would inevitably ‘work’ for some and not for others.

The stark flip-side to all of this, however, was that this place-making was going on in the context of constant existential threat to the organisation, stemming in part from their *not belonging* in the rapidly gentrifying area they had previously occupied, and in part from their lack of funding for a permanent space. Elsewhere, I have explored in depth this ‘mirroring’ of migrant lives and the places of care designed to serve them, arguing that in fact these things are co-constituent (Brenman 2019b). Here, I want to bring out the fact that the move itself, and the events that unfolded in the weeks thereafter, made visible the values held by staff members. Many of these values centred around making people feel they belong, despite a broader politics of exclusion and restricted access to care. But *as well as* being a genuine enactment of these values, the move also made visible the precarious status of places of care in the voluntary sector. It was something I would see play out in various ways across small, community-based services in rapidly changing urban areas like this one; a sign of the transitory, unstable nature of voluntary providers, which increasingly must be movable and ‘adaptable’ to changes in what they can afford and what they are expected to do. In the current system of commissioning clinical services in the UK, charities only get funding for providing the care (in this case, short courses of psychotherapy) and not for what are called ‘core costs’, such as the rent and energy costs needed to maintain a physical building. This put serious constraints on the extent to which values of accessibility, inclusion, and belonging could be brought into being.

Here, we see the dynamic relationships between place, precarity, migration, and belonging unfold. It is at between-moments such as this move that belonging becomes at once valued and destabilised; it comes into sharp focus precisely because it cannot be taken for granted. The focus on the waiting room was salient in many of the visual accounts of accessing care I encountered, and here I have amplified it specifically. As the cliché of liminal spaces goes, it did indeed resemble something of a hotel lobby.^3^ But despite (or more likely because of) this, the waiting room became the new focus for place-making on the part of the clinic staff: children’s toys were arranged in one corner, comfier seats installed, and most significantly, a project introduced where poems were to be projected in multiple languages on a brand new television screen. The waiting room can be seen as a microcosm of in-betweenness or liminality, with people spending short, but often intense and memorable periods of time in this particular space.^4^ Locating analyses in these in-between spaces, as well as moments of transition and change in broader landscapes of care reminds us that even ‘place’ can be unstable and precarious.

## Being in and out of place: precarity as a condition of embodied belonging

What emerged from Mariam and Dayo’s accounts, as well as the broader narrative of the centre as it struggled to make and remake a therapeutic space for their ‘client group’, were important questions about the nature and limits of access and inclusion: What does stability and inclusion mean if you have a mental health problem that can make you feel anything but that? What is inclusion beyond simply a legal or administrative status? If inclusion is also an experience, then what does it take for people who are migrants and suffering mental health problems to experience it? It has also emerged that all of these practices of inclusion and accessing care were inherently precarious. Precarity refers in many ways to vulnerability but pertains less to essential features of the individual than it does to impermanent, often collectivised, states of being. This idea has become widespread in social theory (particularly since Guy Standing’s (2011) sociological account of “the precariat”), as well as global public discourse about anything from the economy to the endangered species (Anna Tsing told us some years ago now that we “hear about precarity in the news everyday” (2015:20)). In this discussion section of the paper, I think with this collective preoccupation, though perhaps in less sweeping terms than in the discussions I have just introduced. I focus specifically on the way precarity emerged from the sociomaterial arrangements of care in UK mental health services.

Moving away from essential or static notions of who ‘is’ precarious, this paper speaks to lines of enquiry that have explored this condition as ‘embodied’ and emergent through encounters with place. Echoing the way precarity emerges in these ethnographic data, Allison (2013:14) has written about the way that “precariousness registers on the senses” in her ethnography of contemporary Japan. Critical scholarship from anthropology and elsewhere in the social sciences has also raised questions about how vulnerabilities are ‘made’, structurally, discursively, or performatively (Quesada 2012). This already takes us a step away from ideas that im/migrants are somehow *inherently* vulnerable, or that this vulnerability is only a mere social category. Thinking through precarity as embodied, in the way I have been demonstrating, performs a similar function. It undoes fixed ideas about people and place (Lien and Melhuus 2007)—or in this case, out-of-placeness—and helps to think through people’s relationships with place in new and changing ways. Neither Dayo nor Mariam’s experiences were straightforwardly about their ‘being migrant’ (from a *different* place) but rather, it was about their encounters with *this particular place* in their very different manifestations of this broad category of being.

The idea of precarity as a shared existential state is not new: Judith Butler has famously suggested that, although the experience of precarity is dependent on the organisation of certain economic and social relations, “no one escapes the precarious dimension of social life” (Butler 2012:148), or, in the words of Berlant, in conversation with these ideas on precarity, “we are all contingent beings” (Berlant, in Puar 2012). Ethnographic attention draws out how this condition becomes more or less visible within changing social worlds. For example, adding empirical weight to the conceptualisation of *precarity in-the-making*, Cabot (2018) has charted the “ongoing precaritization of a [Greek *and* non-Greek] populace that increasingly does not recognise itself ‘at home’”. Similarly, my research opens up an analytical lens to reveal how these issues touch migrants *and* non-migrants (or at least non-self-identified migrants such as Dayo) in similar, different, or surprising ways. What I add to these conversations is an explicit account of the role of materials and spaces in these changing social worlds; in particular, the changing sociomaterial landscape of mental health care in the UK.

None of this is to say that belonging or precarity is experienced in the same way or distributed evenly across people and the places they inhabit. The transience and instability of voluntary care providers I described in the above has been associated with “marginalised services… for socially marginalised people” (Johnsen, Cloke, and May 2005:334). Mariam’s story of temporary or dilapidated accommodation on the edge of town was also a clear demonstration of precarious life outside of the care system—a typical story in the current patterns of the urban resettlement of migrants in the UK (Darling 2017). Neither of these conditions could be said to affect all people or the deeply unequal strata of the care system in the same way. To return to Butler (2012:148), this is more than an unfortunate case of some falling out of their place of security, and arguably a “tactical distribution of precarity… that depends on dominant norms”. What I have been describing, then, is a manifestation of *precarious belonging*^5^, sensed differently across bodies in a particularly unstable place. Senses of belonging and precarity, and their different gradients or distributions, come hand-in-hand. Belonging becomes particularly visible, and valued, when it has been called into question or destabilised—for example, where access has been restricted elsewhere, or a re-location enforced, and a new space of care formed as a result. Only when belonging is taken for granted, does precarity disappear from view.

## Conclusions

This paper has exploited a moment of transition—the moving of a psychotherapy centre from one urban location to another—to explore the notion of embodied belonging. Crucially, this brought the notion into contact with ‘on the ground’ dynamics of a fragmented health care system at a time of austerity, where specific provision for im/migrant communities is uncertain, unstable, and ambivalently supported. These conditions of precarity were described ethnographically, in relation to the situated experiences of two women accessing psychotherapeutic care and those providing this care. Far from being the antithesis of ‘embodied belonging’, precarity emerged as intimately related to the concept. I therefore argue that precarity should be ‘placed’, both within the concept of embodied belonging, and ethnographically, in broader landscapes of care.

Throughout the paper, I have sought to provide a situated analysis of a project to include the excluded, to demonstrate how places participate in and enact (non-)belonging. This sociomaterial approach illuminates how belonging emerges as inherently precarious here. Projects to make and maintain places of inclusion are both motivated *and* disrupted by materially unstable conditions (by a lack of core funding for rent or a state-provided location from which to work). And for clients, the relation between belonging and precarity emerges as a particular function of being ‘placed’ within a mental health care system and the physical manifestation of this placing. This, I argue, provides fresh insights into how projects seeking to achieve belonging through practices of inclusion become complicated, beyond legal and moral questions of access, to the “micro dimensions of therapeutic geographies” (Parkinson and Behrouzan 2015:326).

My focus on the sociomaterialities of place, and the methodological contribution of mapping moments of access, has both disturbed and enriched one element of Mattes and Lang’s analytical concept of ‘embodied belonging’ for this special issue. It aimed to challenge notions of belonging and being ‘in place’ as an abstract category, thinking about how bodies, places, and materials can play into and ‘make up’ these socio-spatial conditions. The ethnographic material disturbs the notion of embodied belonging, in that it denies it stability and complicates what it means to belong or not belong, or to be included or excluded. Yet in doing so, these data also enrich the notion and ground it in current configurations of care, where precarity emerges from bodies and materials assembled in (or out of) place. This has important implications for the way that we understand issues of mental health care access, beyond abstract values and categories of inclusion. In conditions of austerity and increased reliance on extra-state care, ‘placing’ precarity at the centre of this conversation on embodied belonging directs our attention to the material constraints, impermanence, and spatial politics of projects to include the excluded.

## Footnotes

1 I have taken the lead from Sarah Willen and others in adopting the deliberately ambiguous term of ‘im/migrants’ to “acknowledge the tenuous, shifting, and at times illusory nature of the distinction between migrants and immigrants”. (2012a, b:806)

2 The function of mapping, then, was a means to elicit and analyse live accounts of encounters with place, rather than fixed ‘representations’ of a setting. As such, the isolated sections of the maps I reproduce in this paper serve only to be illustrative of the images being generated during the mapping and should not in themselves be viewed as fixed ‘products’.

3 Places designed only for waiting have captured the attention of those interested in health care spaces (Buse and Twigg 2014, for example) and the cultural archetype of the hotel lobby has been drawn upon to highlight the strange liminality of these sites (Tallack 2002, cited in Buse and Twigg 2014).

4 This provides a slightly different view of the waiting room to how it is usually explored anthropologically, with the emphasis on the ‘waiting’ rather than on the ‘room’ itself (Strathmann and Hay 2009; Auyero 2017).

5 ‘Precarious belonging’ is a phrase that resonates in geopolitical analyses (Wang and Goh 2019) but this does not speak to the crucial elements of embodiment and materiality that are central to this ethnographic account, and the series as a whole.

## References

[CR2] Allison Anne (2013). Precarious Japan.

[CR4] Auyero, Javier. 2017. Mothers Matter: Developing the ‘Waiting Mother,’ Somatosphere, September 18th 2017, http://somatosphere.net/2017/mothers-matter-developing-the-waiting-mother.html/

[CR5] Bagnoli Anna (2009). Beyond the Standard Interview: The Use of Graphic Elicitation and Arts-Based Methods. Qualitative Research.

[CR6] Bieler Patrick, Klausner Martina (2019). Niching in Cities under Pressure. Tracing the Reconfiguration of Community Psychiatric Care and the Housing Market in Berlin. Geoforum.

[CR8] Brenman Natassia F. (2019). A Composite Case: Thinking with ‘BME’ Categories in UK Mental Health Care. Medicine Anthropology Theory.

[CR7] Brenman Natassia F (2019). Place, Need and Precarity in UK Mental Health Care: An Ethnography of Access [PhD diss].

[CR9] Buse Christina, Twigg Julia (2014). Looking ‘out of Place’: Analysing the Spatial and Symbolic Meanings of Dementia Care Settings through Dress. International Journal of Ageing and Later Life.

[CR10] Butler Judith (2012). Precarious Life, Vulnerability, and the Ethics of Cohabitation. The Journal of Speculative Philosophy.

[CR11] Cabot Heath (2013). The Social Aesthetics of Eligibility: NGO Aid and Indeterminacy in the Greek Asylum Process. American Ethnologist.

[CR12] Cabot Heath (2017). Philia and Phagia. HAU Journal of Ethnographic Theory.

[CR13] Cabot, Heath. 2018. Reading the Signs: Dust, Smoke, and Displacement in Athens. Allegra Laboritory, http://allegralaboratory.net/reading-the-signs-dust-smoke-and-displacement-in-athens/.

[CR14] Castañeda Heide (2010). Im/Migration and Health: Conceptual, Methodological, and Theoretical Propositions for Applied Anthropology. NAPA. Bulletin, Napa.

[CR15] Darling Jonathan (2017). Forced Migration and the City. Progress in Human Geography.

[CR16] Dennis Fay (2016). Encountering ‘“ Triggers ”’: Drug – Body – World Entanglements of Injecting Drug Use. Contemporary Drug Problems.

[CR17] Fassil, Yohannes, and Angela Burnett. 2015. Commissioning Mental Health Services for Vulnerable Adult Migrants Guidance for Commissioners. London: MIND, https://www.mind.org.uk/media/3168649/vulnerable-migrants_2015_mindweb.pdf.

[CR18] Fassin Didier, Rechtman Richard (2005). An Anthropological Hybrid: The Pragmatic Arrangement of Universalism and Culturalism in French Mental Health. Transcultural Psychiatry.

[CR19] Fassin Didier (2012). Humanitarian Reason: A Moral History of the Present.

[CR20] Gauntlett David, Holzwarth Peter (2006). Creative and Visual Methods for Exploring Identities. Visual Studies.

[CR21] Giordano Cristiana (2014). Migrants in Translation: Caring and the Logics of Difference in Contemporary Italy.

[CR22] Gottlieb Nora, Filc Dani, Davidovitch Nadav (2012). Medical Humanitarianism, Human Rights and Political Advocacy: The Case of the Israeli Open Clinic. Social Science and Medicine.

[CR23] Greco Monica (2004). The Politics of Indeterminacy and the Right to Health. Theory, Culture & Society.

[CR24] Hall Suzanne (2013). Super-Diverse Street: A ‘Trans-Ethnography’ across Migrant Localities. Ethnic and Racial Studies.

[CR25] Health and Social Care Act. 2012. Health and Social Care Act. Chapter 7, http://www.legislation.gov.uk/ukpga/2012/7/contents/enacted.

[CR26] Huschke Susann (2014). Performing Deservingness. Humanitarian Health Care Provision for Migrants in Germany. Social Science & Medicine.

[CR27] Ingold Tim (2006). Up, across and Along. Place and Location: Studies in Environmental Aesthetics and Semiotics.

[CR28] Johnsen Sarah, Cloke Paul, May Jon (2005). Transitory Spaces of Care: Serving Homeless People on the Street. Health and Place.

[CR29] Kareem Jafar, Littlewood Roland (2000). Intercultural Therapy.

[CR30] Kirmayer Laurence, Narasiah L., Munoz M., Rashid M., Ryder A.G., Guzder J., Hassan G., Rousseau C., Pottie K. (2011). Common Mental Health Problems in Immigrants and Refugees: General Approach in Primary Care. Canadian Medical Association Journal.

[CR31] Lähdesmäki Tuuli, Saresma Tuija, Hiltunen Kaisa, Jäntti Saara, Sääskilahti Nina, Vallius Antti, Ahvenjärvi Kaisa (2016). Fluidity and Flexibility of ‘Belonging’: Uses of the Concept in Contemporary Research. Acta Sociologica (United Kingdom).

[CR32] Larchanché Stéphanie (2012). Intangible Obstacles: Health Implications of Stigmatization, Structural Violence, and Fear among Undocumented Immigrants in France. Social Science and Medicine.

[CR33] Latour Bruno (1987). Science in Action: How to Follow Scientists and Engineers through Society.

[CR34] Law John (2004). After Method: Mess in Social Science Research.

[CR35] Lien Marianne E. (2015). Roots, Rupture and the Tasmanian Lives of the Monterey Pine. Journal of Material Culture.

[CR36] Lien Marianne, Melhuus Marit (2007). Holding Worlds Together: Ethnographies of Knowing and Belonging.

[CR37] Lipsedge Maurice, Littlewood Roland (2005). Aliens and Alienists: Ethnic Minorities and Psychiatry.

[CR38] Literat Ioana (2013). ‘A Pencil for Your Thoughts’: Participatory Drawing as a Visual Research Method with Children and Youth. International Journal of Qualitative Methods.

[CR39] Matza Tomas (2018). Shock Therapy: Psychology, Precarity, and Well-Being in Postsocialist Russia.

[CR40] McGrath Laura, Reavey Paula (2013). Heterotopias of Control: Placing the Material in Experiences of Mental Health Service Use and Community Living. Health and Place.

[CR41] Moser Ingunn, Nord Catherina, Högström Ebba (2017). Sustaining the Webs of Life: An STS Approach to Space, Materiality and Subjectivity in Care. Caring Architecture: Institutions and Relational Practices.

[CR42] Namer Yudit, Oliver Razum (2018). Settling Ulysses: An Adapted Research Agenda for Refugee Mental Health. International Journal of Health Policy and Management.

[CR43] Nellums, Laura, Kieran Rustage, Sally Hargreaves, Jon Friedland, Miller Anna, Hiam Lucinda, Preeti Kathrecha, Rosie Wallbank, and Suzanne Devlin. 2018. Access to Healthcare for People Seeking and Refused Asylum in Great Britain: A Review of Evidence. *Equality and Human Rights Commission Research Report Series*. https://www.equalityhumanrights.com/sites/default/files/research-report-121-people-seeking-asylum-access-to-healthcare-evidence-review.pdf, accessed June 2020.

[CR45] Ootes Sabina, Pols Jeannette, Tonkens Evelien, Willems Dick (2013). Opening the Gift: Social Inclusion, Professional Codes and Gift-Giving in Long-Term
Mental Healthcare. Culture, Medicine & Psychiatry.

[CR47] Ootes Sabina, Pols Jeannette, Tonkens Evelien, Willems Dick (2013). Where Is the Citizen? Comparing Civic Spaces in Long-Term Mental Healthcare. Health & Place.

[CR44] Parkinson Sarah E., Behrouzan Orkideh (2015). Negotiating Health and Life: Syrian Refugees and the Politics of Access in Lebanon. Social Science and Medicine.

[CR46] Parr Hester (2000). Interpreting the ‘Hidden Social Geographies ’ of Mental Health: Ethnographies of Inclusion and Exclusion in Semi-Institutional Places. Health & Place.

[CR49] Pols Jeannette (2016). Analyzing Social Spaces: Relational Citizenship for Patients Leaving Mental Health Care Institutions. Cross-Cultural Studies in Health and Illness.

[CR50] Puar Jasbir (2012). Precarity Talk: A Virtual Roundtable with Lauren Berlant, Judith Butler, Bojana Cvejić, Isabell Lorey, Jasbir Puar, and Ana Vujanović. TDR The Drama Review.

[CR51] Quesada James (2012). Illegalization and Embodied Vulnerability in Health. Social Science and Medicine.

[CR53] Sargent Carolyn (2012). Special Issue Part I: ‘Deservingness’ and the Politics of Health Care. Social Science and Medicine.

[CR55] Standing Guy (2011). The Precariat: The New Dangerous Class.

[CR56] Strathmann Cynthia Miki, Hay Cameron (2009). Working the Waiting Room: Managing Fear, Hope, and Rage at the Clinic Gate. Medical Anthropology.

[CR57] Stevenson Lisa (2014). Life Beside Itself: Imagining Care in the Canadian Arctic.

[CR58] Tallack Douglas, Leach Neil (2002). ‘Waiting, Waiting’: The Hotel Lobby, in the Modern City. The Hieroglyphics of Space: Reading and Experiencing the Modern Metropolis.

[CR59] Thrift Nigel (2004). Movement-Space: The Changing Domain of Thinking Resulting from the Development of New Kinds of Spatial Awareness. Economy and Society.

[CR60] Ticktin Miriam (2006). Where Ethics and Politics Meet: The Violence of Humanitarianism in France. American Ethnologist.

[CR61] Tsing Anna L. (2015). The Mushroom at the End of the World: On the Possibility of Life in Capitalist Ruins.

[CR62] Vertovec Steven (2007). Super-Diversity and Its Implications. Ethnic and Racial Studies.

[CR63] Wang Chih-Ming, Goh Daniel P.S. (2019). Precarious Belongings: Affect and Nationalisms in Asia.

[CR64] White Paul, Hillman Alexandra, Latimer Joanna (2012). Ordering, Enrolling, and Dismissing: Moments of Access across Hospital Spaces. Space and Culture.

[CR67] Willen Sarah S. (2011). Do ‘Illegal’ Im/Migrants Have a Right to Health? Engaging Ethical Theory as Social Practice at a Tel Aviv Open Clinic. Medical Anthropology Quarterly.

[CR65] Willen, Sarah S. 2012a. Migration, ‘Illegality,’ and Health: Mapping Embodied Vulnerability and Debating Health-Related Deservingness. Social Science and Medicine 74(6):805–811. 10.1016/j.socscimed.2011.10.041. 10.1016/j.socscimed.2011.10.041 22257746

[CR66] Willen, Sarah S. 2012b. How Is Health-Related ‘Deservingness’ Reckoned? Perspectives from Unauthorized Im/Migrants in Tel Aviv. Social Science and Medicine 74(6): 812–821. 10.1016/j.socscimed.2011.06.033. 10.1016/j.socscimed.2011.06.033 21821324

[CR68] Yuval-Davis Nira, Wemyss Georgie, Cassidy Kathryn (2017). Everyday Bordering, Belonging and the Reorientation of British Immigration Legislation. Sociology.

